# Perceptions of a food benefit programme that includes financial incentives for the purchase of fruits and vegetables and restrictions on the purchase of foods high in added sugar

**DOI:** 10.1017/S1368980021001051

**Published:** 2022-06

**Authors:** Fatima A Fagbenro, Tessa Lasswell, Sarah A Rydell, J Michael Oakes, Brian Elbel, Lisa J Harnack

**Affiliations:** 1 School of Public Health, Division of Epidemiology and Community Health, University of Minnesota Twin Cities, Minneapolis, MN 55455, USA; 2 Department of Population Health, New York University Wagner, New York City, NY, USA

**Keywords:** Financial incentives and restrictions, Supplemental Nutrition Assistance Program (SNAP), Policy change strategies, Poor diet quality

## Abstract

**Objective:**

To report perspectives of participants in a food benefit programme that includes foods high in added sugar (FAS) restrictions and FAS restrictions paired with fruits and vegetables (F/V) incentives.

**Design:**

Randomised experimental trial in which participant perspectives were an exploratory study outcome.

**Setting:**

Participants were randomised into one of three Supplemental Nutrition Assistance Program (SNAP)-like food benefit programme groups: (1) restriction: not allowed to buy FAS with benefits; (2) restriction paired with incentive: not allowed to buy FAS with benefits and 30 % financial incentive on eligible F/V purchased using benefits; or (3) control: same food purchasing rules as SNAP. Participants were asked questions to assess programme satisfaction.

**Participants:**

Adults in the Minneapolis-St. Paul, MN metropolitan area, eligible for but not currently participating in SNAP who completed baseline and follow-up study measures (*n* 254).

**Results:**

Among remaining households in each group, most found the programme helpful in buying nutritious foods (88·2 %–95·7 %) and were satisfied with the programme (89·1 %–93·0 %). Sensitivity analysis results indicate that reported helpfulness and satisfaction with the programme may in some instances be lower among the restriction and the restrictions paired with incentive groups in comparison to the control group.

**Conclusions:**

A food benefit programme that includes restriction on purchase of FAS or restriction paired with a financial incentive for F/V purchases may be acceptable to most SNAP-eligible households with children.

The Supplemental Nutrition Assistance Program (SNAP), formerly known as The Food Stamp Program, was implemented in 1964 and is the largest federal aid programme in the USA. The programme was designed to address food insecurity among low-income households via provision of funds for the purchase of food. In 2019, about 36 million Americans received food assistance to the cost of $60 billion^(1)^. Recent studies have suggested that despite the effectiveness of SNAP at reducing food insecurity among participants, participating in SNAP is associated with unhealthful dietary habits^(2–5)^ and a higher rate of obesity among participants compared to income-eligible non-participants^(6,7)^.

SNAP, unlike other federal food assistance programmes, has permissive rules on the types of foods that may be purchased using programme benefits, with a limited number of food items (e.g. alcohol, hot foods, and dietary supplements) classified as ineligible for purchase using SNAP benefits. These permissive rules have been criticised and have triggered questions on whether SNAP benefits are being utilised to excessively consume unhealthful foods which may contribute to higher rates of obesity and other diet-related conditions such as diabetes and CVD^(8)^.

In response to these coexisting public health issues of food insecurity and poor nutrition, several changes to SNAP are being considered. One is prohibiting the use of SNAP benefits to purchase foods high in added sugar (FAS) and another is offering an incentive for the purchase of fruits and vegetables (F/V)^(9)^. Participant perspectives on these strategies are important to understanding the potential benefits and adverse consequences or concerns that may need to be addressed in advance of any programme changes. Currently, there is limited information on participant perspectives. Data from prior studies have been obtained via surveys that asked SNAP participants to speculate on acceptance/support of these potential programme changes^(10–12)^. Just two experimental trials have assessed perspectives in the context of the lived experience of participating in a food benefit programme with these programme features in place^(13,14)^. The US Department of Agriculture (USDA) Healthy Incentives Pilot study explored satisfaction with F/V financial incentives among SNAP participants^(13)^. A study by Rydell *et al.* investigated satisfaction with a SNAP-like food benefit programme with F/V financial incentives and/or restrictions^(14)^. Participants in this study included people who were eligible for SNAP but not participating or near eligible for the programme.

Further insight into the acceptance of these strategies is necessary, especially from participants in food benefit programmes that have implemented these strategies. Consequently, the purpose of this paper is to report the perspectives of those participating in a randomised trial in which food purchasing rules and incentives varied across experimental groups (Clinical Trial Registration NCT03363048). Programme satisfaction and perceived effects on food intakes were exploratory outcomes in this study in which diet quality of adult and child participants was the primary outcome.

## Methods

**Fig. 1:**
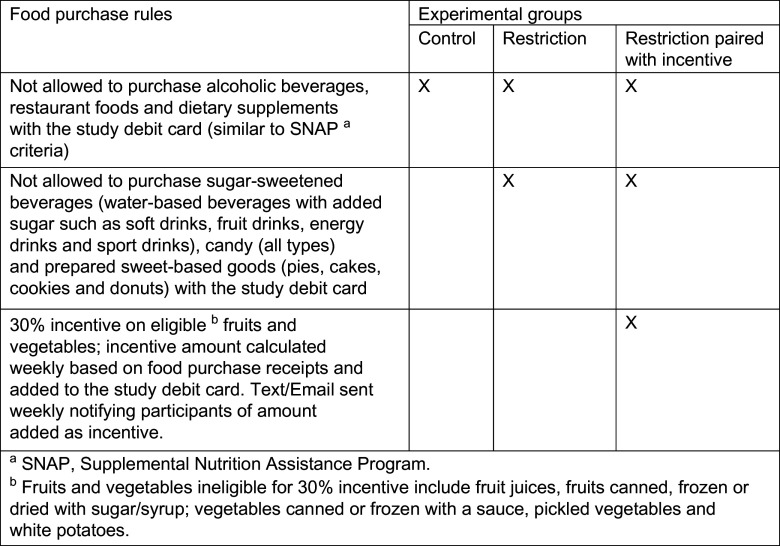
Description of the three experimental groups to which participants in the GAPS for Families study were randomly assigned. GAPS, Grocery Assistance Program Study for Families

Data reported in this paper were collected as part of the Grocery Assistance Program Study for Families (GAPS for Families), an experimental trial designed to evaluate the effects on family nutrition of: (1) restricting the use of food programme benefits for purchasing sugar-sweetened beverages, sweet-baked goods and candies (FAS) and (2) implementing restrictions paired with a 30 % financial incentive for F/V purchases made using programme benefits. To test these research questions, households eligible for SNAP, but not currently participating, were randomised to one of the following food benefit programme groups: (1) restriction on use of food programme benefits for purchasing FAS; (2) restrictions paired with 30 % financial incentive for F/V purchased with programme benefits; or (3) control group which has the same food purchasing rules as SNAP. Further detail regarding food purchase rules for each group is provided by Figure 1.

During the intervention period, all study participants were provided with a study debit card to which funds for food purchasing were added every 4 weeks for a 20-week period. The amount of funds added every 4 weeks was determined using similar household information and formula used by the Minnesota Department of Health and Human Services to determine benefit levels for SNAP participants in Minnesota. Those in the restriction group were given rules to not purchase sugar-sweetened beverages, sweet-baked goods, or candies with the study debit card. Out-of-pocket funds could still be used to purchase these types of foods. In the restriction paired with incentive group, participations had the same restriction paired with incentive of 30 % cash back on their study debit card for F/V purchased using the debit card that were eligible for the incentive. For example, if $10 worth of eligible F/V were purchased using the study debit card, $3 was added to the card for use in making future food purchases. Compliance with purchasing rules and calculation of the incentive amount for those in the restriction paired with incentive group were determined through a combination of food purchase receipts submitted by study participants throughout the experimental period and expenditure information available to the research team from the transaction history provided by the debit card vendor. The submitted receipts were reviewed to assess compliance with food purchasing rules. Repeated non-compliance or flagrant violations of purchasing rules resulted in discontinuation of receipt of food programme benefits.

### Study sample

The aim was to recruit 240 households with young children that were eligible but not participating in SNAP at the time of study enrolment. Recruitment took place in the Minneapolis/St Paul, MN metropolitan area, on an ongoing basis over the study enrolment period (rolling enrolment). Recruitment had a multi-pronged approach utilising referrals from community agencies that assist low-income households in enrolling in government assistance programmes such as SNAP, in-person recruitment at community events, advertising on Craigslist, higher education institution student-parent groups, and posting study information on social media platforms such as Facebook and NextDoor.

Study eligibility criteria included: (1) not currently participating in SNAP and willing to defer enrolment in the programme for 5 months (until end of experimental period); (2) meet income and other eligibility requirements for SNAP in Minnesota; (3) have a child aged 3–11 years of age living in the household (if household has more than one child in this age range, one was selected randomly for inclusion in study); and (4) have a main food shopper who is able to speak and understand English or Spanish.

### Data collection

A variety of study measures were collected during baseline and follow-up measurement periods. The baseline measurement period, which was conducted over approximately a 2-week period, included an in-person visit during which study eligibility was confirmed, consent and child assent were obtained, and the adult participant was asked to complete a questionnaire. The questionnaire included questions on demographics, health history, past and present participation in food assistance programmes, and household food security (US Household Food Security Survey Module: Three-Stage Design, with Screeners modified to ask about the past 30 d). Study staff measured height and weight of both the adult and child during in-person visits. Anthropometric measurements were collected using standard protocols, including height measured to the nearest 0·1 cm and weight measured to the nearest 0·1 kg. BMI was calculated as weight in kilograms divided by height in metres squared (kg/m^2^). As a measure of food and nutrient intake, three unannounced telephone-administered 24-h dietary recalls were collected from both the adults and child participants in each household at each time period.

Follow-up measures were collected predominately between weeks 13 and 16 of the 20-week experimental period, although some participants completed measures in 17–20 weeks. Measures collected during this period included another three telephone-administered 24-h dietary recalls for each child and adult participant, along with an in-person visit during which the height and weight of the adult and child participants were measured using the same procedures as baseline. A self-administered questionnaire was also provided.

The follow-up questionnaire included a series of open- and close-ended question to assess satisfaction, acceptability, and perceived usefulness of the food benefit programme to which they had been randomised. Close-ended questions asked of all participants included: (1) How helpful was the study food benefit programme in buying enough food for your household? (2) How helpful was the study food benefit programme in buying healthful/nutritious foods for your household? (3) How helpful was the study food benefit programme in buying the kinds of foods you want to purchase? and (4) Overall how satisfied were you with the study food benefit programme?

Open-ended questions asked only of participants in the restriction and restriction paired with incentive groups included: (1) You are not allowed to buy some sugary foods such as soft drinks, candies and cookies with your study food benefits. What do you think of these rules? (2) Do you think you are purchasing fewer sugary foods because they are not allowed to be purchased with the study debit card? If yes, does this affect your household in any way? If yes, how? Participants in the restriction paired with incentive group were also asked: (1) You were given a bonus for purchasing F/V with the study debit card. What do you think of these rules? (2) Does the bonus make you want to purchase more vegetables than you otherwise would? If yes, in what way? If no, why not? (3) Does the bonus make you want to purchase more fruits than you otherwise would? If yes, in what way? If no, why not?

### Statistical analysis

Participants who completed the questionnaire administered during the follow-up study measurement period were included in the analyses reported in this paper. Quantitative analyses were performed for responses to close-ended questions using the SAS statistical software (version 9.4.). Means and frequencies were calculated to describe the study sample and responses to close-ended questions. Logistic regression analyses were conducted to estimate the degree to which reported programme helpfulness and satisfaction differed between the restriction and restriction paired with incentive groups in comparison to the control (reference) group. Responses to each question about programme helpfulness and satisfaction were dichotomised for these models. Covariates included in the models included age of adult, race of adult (Black/White/other), baseline food security status and baseline BMI category of adult. To explore potential bias due to unequal follow-up losses, two sensitivity analyses were carried out. In one approach, sensitivity to missing data (13 % for each outcome) was examined by multiple imputation (30 imputations using all model covariates and outcomes). In the other approach, all of those lost to follow-up were imputed to have a negative response to questions about programme helpfulness and satisfaction.

Qualitative analysis of responses to open-ended questions was performed by two reviewers using a direct thematic analysis approach. The open-ended data were entered into Microsoft Excel and given a cursory read by two of the authors (FF and LH) to identify themes for coding. These open-ended data were then copied into two Microsoft Excel spreadsheets and independently reviewed by the two of the authors (FF and TL) to refine the coding scheme and carry out coding. Coding was carried out independently by FF and TL in Microsoft Excel. In the cases where there was a coding discrepancy between the two independent coders, the responses were flagged and discussed until consensus was reached.

## Results

**Fig. 2:**
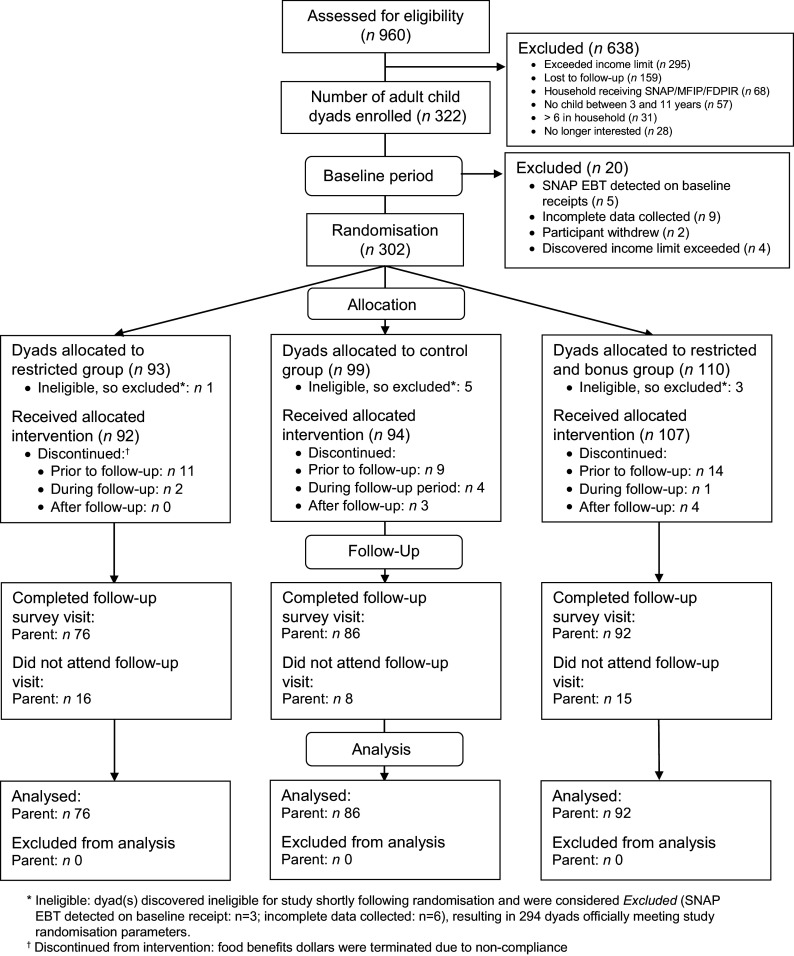
Flow of study participants in the GAPS for Families experimental trial. GAPS, Grocery Assistance Program Study for Families; SNAP, Supplemental Nutrition Assistance Program

Study participants were recruited in 13 waves, from May 2018 to October 2019, with a total of 322 adult/child dyads enrolled in the study (Fig. 2). Among these dyads, 302 completed baseline measurements required for randomisation and were randomised to an experimental group. Overall, 254 completed the questionnaire administered at follow-up and were therefore included in the analyses reported in this paper. Follow-up rates varied across the experimental groups, with loss to follow-up higher in the restriction and restriction plus incentive groups (23 % and 25 %, respectively) compared to the control group (11 %).

The mean age of adult participants was 35·2 (sd 7·85) years, with no significant difference by the experimental group (*P* = 0·72). Other demographic data of the adult study participants are presented disaggregated by experimental groups (Table 1). Most of the participants were female, overweight or obese, and living in a household with low or moderate child food security at baseline. Food security status was significantly different between experimental groups (*P* = 0·007).

**Table 1:** Baseline demographic and household characteristics of adult participants in the GAPS for Families study with a completed follow-up survey (*n* 254)

Characteristics	Total		Control		Restriction		Restriction paired with incentive		*P*-value
	%	*n*	%	*n*	%	*n*	%	*n*	
Sex									
Female	92·5	235	93·0	80	90·8	69	93·5	86	0·79
Male	7·5	19	7·0	6	9·2	7	6·5	6	
Ethnicity									
Hispanic/Latino	10·6	27	10·5	9	10·5	8	10·9	10	0·996
Non-Hispanic/Latino	89·4	227	89·5	77	89·5	68	89·1	82	
Race									
Black	35·0	89	41·9	36	32·9	25	30·4	28	0·70
White	45·3	115	38·4	33	46·1	35	51·1	47	
Multi-racial	7·9	20	7·0	6	9·2	7	7·6	6	
Other	11·8	30	12·8	11	11·8	9	10·9	10	
Marital status									
Single	69·3	176	73·3	63	68·4	52	66·3	61	0·59
Married or partnered	30·7	78	26·7	23	31·6	24	33·7	31	
Household size									
2	14·2	36	15·1	13	13·2	10	14·1	13	0·66
3	26·4	67	26·7	23	25·0	19	27·2	25	
4	28·0	71	25·6	22	26·3	20	31·5	29	
5	21·7	55	18·6	16	25·0	19	21·7	20	
6 or more	9·8	25	14·0	12	10·5	8	5·4	5	
Education level									
High school graduate or less	24·0	61	26·7	23	30·3	23	16·3	15	0·13
Some college or associate degree	51·2	130	46·5	40	52·6	40	54·4	50	
College graduate or higher	24·8	63	26·7	23	17·1	13	29·4	27	
Food security status of children in household									
Low food security	10·2	26	8·1	7	10·5	8	12·0	11	0·007
Moderate food security	52·8	134	51·2	44	68·4	52	41·3	38	
High food security	36·6	93	40·7	35	21·1	16	45·7	42	
Body weight status of adult participants									
Normal or underweight (BMI < 25)	19·3	49	23·3	20	18·4	14	16·3	15	0·07
Overweight (BMI 25–29·9)	16·1	41	22·1	19	11·8	9	14·1	13	
Obese (BMI ≥ 30	58·3	148	52·3	45	65·8	50	57·6	53	

GAPS, Grocery Assistance Program Study for Families.

### Helpfulness and satisfaction with the study food benefit programmes

Table 2 shows the response to the questions asked about satisfaction with the experimental group to which they were randomised. Most participants in each of the three experimental groups found the programme helpful for purchasing enough food (87·0 %–89·5 %), healthful/nutritious foods (88·2 %–95·7 %) and desired foods (81·6 %–92·4 %) for their households. In addition, most participants in each group reported they were satisfied with the programme (89·1 %–93·0 %). Results from logistic regression analyses controlling for baseline measures of adult age, adult race, baseline child food security status and adult body weight status indicated that level of satisfaction did not vary significantly between groups (Table 3). However, results from sensitivity analyses using two imputation methods (Table 3) suggest those in the restriction group may be less likely to report the programme to be helpful in buying healthful/nutritious foods and the kinds of foods the household want in comparison to the control group. In addition, results from sensitivity analyses suggest those in the restriction paired with incentive group may be less likely to report being satisfied with the programme in comparison to the control group.

**Table 2:** Frequency of responses to questions asked about the helpfulness of the programme and level of satisfaction with the programme by experimental group, GAPS for Families (*n* 254)

	Control (*n* 86)		Restriction (*n* 76)		Restriction paired with incentive (*n* 92)	
	%	*n*	%	*n*	%	*n*
Helpfulness in buying enough food for household						
Helpful	89·5	77	89·5	68	87·0	80
Somewhat helpful	10·5	9	9·2	7	12·0	11
Not helpful	0	0	1·3	1	1·1	1
Helpful in buying healthful/nutritious foods						
Helpful	95·3	82	88·2	67	95·7	88
Somewhat helpful	4·7	4	11·8	9	3·3	3
Not helpful	0	0	0	0	1·1	1
Helpful in buying the kinds of foods you want						
Helpful	90·7	78	81·6	62	92·4	85
Somewhat helpful	9·3	8	18·4	14	6·5	6
Not helpful	0	0	0	0	1·1	1
Satisfaction with the study food benefit programme						
Satisfied	93·0	80	90·8	69	89·1	82
Somewhat satisfied	7·0	6	7·9	6	9·8	9
Somewhat dissatisfied	0	0	1·3	1	0	0
Dissatisfied	0	0	0	0	1·1	1

GAPS, Grocery Assistance Program Study for Families.

**Table 3:** Odds* of reporting the food benefit programme to be helpful and reporting satisfaction with the food benefit programme by experimental group (Odd Ratios values and 95 % CI)

	Restriction group *v*. control (reference) group						Restriction paired with incentive group *v*. control (reference) group					
	Observed (*n* 254)		Imputation method 1† (*n* 293)		Imputation method 2‡ (*n* 293)		Observed (*n* 254)		Imputation method 1† (*n* 293)		Imputation method 2‡ (*n* 293)	
	OR	95 % CI	OR	95 % CI	OR	95 % CI	OR	95 % CI	OR	95 % CI	OR	95 % CI
Helpfulness in buying enough food for household§	1·01	0·33, 3·12	1·42	0·81, 2·5	0·57	0·27, 1·20	0·86	0·30, 2·49	0·64	0·39, 1·06	0·60	0·29, 1·23
Helpful in buying healthful/nutritious foods§	0·37	0·10, 1·36	0·56	0·29, 1·09	0·35	0·16, 0·77	1·44	0·29, 7·09	0·75	0·37, 1·50	0·61	0·27, 1·39
Helpful in buying the kinds of foods you want§	0·47	0·18, 1·28	0·52	0·31, 0·86	0·38	0·18, 0·78	1·33	0·42, 4·19	0·98	0·56, 1·72	0·73	0·34, 1·53
Satisfaction with the study food benefit programme||	0·72	0·21, 2·47	1·14	0·61, 2·12	0·46	0·21, 1·02	0·54	0·16, 1·84	0·39	0·22, 0·69	0·47	0·22, 1·02

* Results from multivariate logistic regression models that included that the following covariates: age of adult, race of adult (Black/White/other), baseline food security status and baseline BMI category of adult.

† Multiple imputation (30 imputations using all model covariates and outcomes) for those lost to follow-up.

‡ All of those lost to follow-up imputed to have a negative response to questions about programme helpfulness and satisfaction.

§ Variable dichotomised for analysis as *Helpful v*. *Somewhat helpful/Not helpful*.

|| Variable dichotomised for analysis as *Satisfied v*. *Somewhat satisfied/Somewhat dissatisfied/Dissatisfied*.

### Participant perspectives on restrictions

#### Insights on restriction from those in the restriction group (76) and the restriction paired with incentives group (92)

When asked what they thought of the programme rule that they could not buy FAS with programme benefits, most of the participants in the restriction and the restriction paired with incentives groups endorsed the rule to some extent, with supportive responses ranging from modest (‘it was ok’) to strongly supportive (‘it was great’). For example, a participant said, *‘I thought they were fair rules! Of course, we all have cravings for these things, but it was such a relief to not have to worry about buying healthy food for my family and where the money was coming from’.*

The primary reason given by participants for endorsing the rules related to the benefit of supporting healthy eating, lifestyle choices and improved health. For example, a restriction group participant said, *‘These were great rules, they enabled me to keep away from less nutritious foods’*, and a restriction paired with incentives group participant said, *‘Great rules. Helps keep clean eating on your mind’*. Another participant said, *‘I think it’s great and encourages more thoughtful/healthy purchases’*.

A few participants stated that they disliked the rules. Reasons for disliking the rules included feeling they were unfair (most frequently mentioned reason) or difficult to adhere to. For example, a restriction group participant said, *‘It’s kind of unfair because the juice we buy is not 100 % so can’t buy it. [CHILD NAME] also wanted candies and I had to tell him no a couple of times’*, and a restriction paired with incentives group participant said, *‘I think that it is difficult some times because of your children if you shop with them, they like to have a snack from time to time. Soft drinks are always a treat’*.

Some within the restriction group expressed understanding of the rationale for the rules but still wanted to be permitted to purchase the restricted items. For example, a participant said, *‘I don’t like it because occasionally I want a sugary item’*.

Two additional themes were identified from the responses of both groups about their thoughts on the restrictions. They include concern about the impact of limiting sugar intake, especially since these were households with children. For example, a restriction group participant said, *‘It was a little hard, as my daughter needs Gatorade as part of her diet for her health, per the Doctor’*. Another participant from the restriction paired with incentives group said, *‘Somewhat unrealistic when you have children. It should be okay to buy it occasional’*.

Inconvenience of the rules was the other theme that emerged. For example, restriction group participants said, *‘It was great, but there were sometimes that I wanted to purchase one’* and *‘It’s hard to remember at the store while shopping never remember the list’*. The restriction paired with incentives group participants also said, *‘It was hard trying to find non-sugar. Sugar is in almost everything’* and *‘It’s sometimes confusing as to what qualifies as a sugary food’*.

#### Perceived impact of restrictions on food purchases

Participants in the restriction and restriction plus incentive groups were asked if the restriction had an impact on their purchasing of FAS (yes/no response option). Among those in the restriction-only group 64 % (*n* 49) stated that the restriction had reduced their purchasing of FAS, of these 51 % (*n* 25) further confirmed that the changes caused their households to consume fewer of the restricted foods.

Among those in the restriction plus incentive group, 76 % (*n* 71) stated that their purchasing habit was affected by the restrictions, and of these 54 % (*n* 38) responded affirmatively when asked if it affects their household in any way. Themes identified on the ways people described how their households were affected included positive behaviour changes (e.g. reduced consumption of unhealthful foods and reduced purchasing temptations) and improved health. For positive behaviour changes, one participant said, *‘Encouraged healthier options stopped drinking pop all together less candy around the house’*. Example of health benefits described by the participants include *‘Good effect!!! Less sugary foods for kids and diabetic husband, helped me keep focused!!!’*, *‘Less cavities’*, *‘We are getting healthier and more fit. I think more about healthier food as well’*, and *‘I have low blood sugar’*. To a lesser extent, participants described some negative effects on their households. They described having concerns about limiting their sugar intake and the emotional turmoil that could come with a sugar detoxification, especially for the children. One participant said, *‘Sugar detox is emotional’*. Another said, *‘The kids have a hard time without sugary foods’*.

#### Participant perspectives on F/V incentive

Participants in the restriction paired with incentive group were asked if the incentive led them to purchase more vegetables than they otherwise would have. Most (73 %) said that the incentive increased their vegetable purchase. When asked ‘In what way?’ the primary reason given was affordability because the incentive gave them the opportunity to buy more vegetables. For example, one participant said, ‘*It allows us to buy even more vegetables to stretch out the money’*. The second most cited reason for the increase in their vegetable purchase was motivation, getting the incentive encouraged purchase of vegetables. For example, one participant said, *‘It’s just encouraging. I want to anyways, but this is just a little push’.* For the small proportion of participants who stated that their vegetable purchase did not change, the most common theme was that they already had adequate vegetable purchasing prior to participating in the study. For example, one participant said, *‘I purchase what I need on how we eat as a family and what we like. We eat pretty healthy’*.

When asked if the incentive led them to purchase more fruit than they otherwise would have, most (66 %) reported responded affirmatively. When asked ‘In what way?’ the primary reason was that the incentive made fruits more affordable. For example, one participant said, *‘We have a little extra to keep buying them’*.

Those who reported no change in their fruit purchase tended to report that they already ate healthy. Two participants identified avoiding food wastage as the reason for the lack of change in fruit consumption for their households. They said, *‘Don’t want fruits to be wasted/spoil’* and *‘I only buy what me & family will eat, don’t want to waste food and have to throw away when it goes bad’*.

## Discussion

This study’s findings confirm positive opinions and support of SNAP participants on the inclusion of financial incentives for F/V purchase with benefit funds identified in previous studies^(11–15)^. For example, the USDA Healthy Incentives Pilot, which provided SNAP participants with 30 % financial incentive bonus for F/V purchase, found that 95 % of the participants endorsed the introduction of the financial incentive strategy^(13)^. Similarly, a study exploring the perception of Californian SNAP participants also found that over 90 % of SNAP participants endorsed the idea of adding an incentive for F/V purchase^(11)^.

Concerns have been raised that restricting the use of programme benefits for purchasing FAS could be perceived as demeaning and unfair to SNAP participants^(16)^. However, findings from the present study and previous studies suggest that a modest majority of SNAP participants are receptive to restrictions on the purchase of FAS^(10–12,14)^. A previous study (carried out by members of the present research team) found that 58 % of participants in a SNAP-like food benefit programme with restrictions or restrictions paired with incentives endorsed the restriction. However, it is important to note that participants in that study included households not eligible for SNAP (households that were ‘near eligible’ and eligible but not participating in SNAP were included), and thus findings may not generalise to SNAP-eligible participants^(14)^. Another study found in a survey of SNAP participants that 75 % endorsed the idea of imposing a restriction on the purchase of sugary beverages with benefit funds^(11)^.

Previous studies have also found that participants are more accepting of the restrictions when paired with incentives for F/V^(11,12)^. For example, Long et al found that nearly half the participants who initially opposed the idea of imposing restrictions decided to support it if it was paired with the financial incentives for F/V^(12)^. Findings from the present study align with these findings as most participants in the restriction group expressed support for purchase restrictions, whereas nearly all participants in the restriction paired with incentive group expressed support of restrictions.

Our results also indicate that participants endorsed these strategies (the incentives for F/V and restriction on FAS) primarily because of the impact on their food intake and health. However, study findings regarding the effect of restrictions on FAS and restrictions paired with F/V incentives on diet quality (primary study outcome) do not support participants’ perceived benefit on food intake (unpublished results).

The participants who disliked the restrictions cited unfairness, compliance difficulty and the potential effect of sugar detoxification as their reasons for their opposition. Addressing these concerns could facilitate acceptance of this type of programme change. For example, allowing a limited fraction of benefits to be used for purchasing FAS rather than none might result in greater acceptance or pairing F/V incentives with restrictions. Or, other strategies could be explored such as providing incentives on a greater range of healthful foods while implementing disincentives for the purchase of FAS.

### Strengths and limitation

Strengths of this study include its experimental design in which participants are reporting the lived experience of purchasing food with a food benefit programme that includes restrictions and restrictions paired with incentives. In contrast, most previous studies were surveys that relied on attitudes and beliefs on proposed (theoretical) SNAP programme modifications^(10–12)^.

The study limitations include the sample representativeness. Though the study sample included SNAP-eligible families with young children, it is possible the sample does not represent those who participate in the programme, given that these families had not already enrolled in the programme. Also, the sample was recruited from one metropolitan area and therefore may not reflect the general SNAP population. Our findings also do not capture perception of non-English or Spanish speakers because the surveys and instructions were provided English and Spanish only. It is important to note that the high rate of positive appraisal of restrictions and incentives by the participants could be attributable in part to social desirability bias and loss to follow-up. Close to 25 % of participants randomised to the restriction and restriction paired with incentive groups were lost to follow-up, and those participants may have had less favourable views of restrictions and incentives compared to those who completed follow-up measures.

### Conclusions

This study assesses participant satisfaction and acceptance with restricting the purchase of FAS in a food benefit programme, and imposing these restrictions paired with a financial incentive for the purchase of F/V. Findings suggest that the inclusion of an incentive for F/V purchase and imposing restrictions on the purchase of FAS in a food benefit programme like SNAP may be acceptable to most programme participants. This information may be useful to policymakers as they consider ways to reshape SNAP, so that it better supports family nutrition.
